# Microstructural Evolution of the Rail Steels Manufactured by Hanyang Iron Works

**DOI:** 10.3390/ma15165488

**Published:** 2022-08-10

**Authors:** Tinghui Man, Yihao Zhou, Nan Dong, Tengshi Liu, Han Dong

**Affiliations:** 1 School of Materials Science and Engineering, Shanghai University, Shanghai 200444, China; 2 Zhejiang Institute of Advanced Materials, Shanghai University, Jiashan 314113, China

**Keywords:** Hanyang Iron Works, rail steels, microstructural evolution, EBSD

## Abstract

The microstructural evolution of the rail steels manufactured by Hanyang Iron Works was investigated through optical microscopy (OM), scanning electron microscopy (SEM) and electron backscattered diffraction (EBSD). The OM and SEM images reveal that the microstructures are ferrite with a small amount of pearlite in the rail steel 1904, reticular ferrite and pearlite in the rail steels 1911 and 1921, and full pearlite in the rail steels 1917 and 1919, respectively. The EBSD results show that the rail steel 1904 holds the smallest average grain diameter owing to the pearlite with small size. Moreover, the average grain diameter of the pearlite cluster decreases in the rail steels manufactured after 1908, except for 1921, when it was on the verge of bankruptcy. The rail steels 1917 and 1919 exhibit a higher proportion of low angle grain boundaries and local misorientation with angles lower than 1°. Besides, the grain boundary misorientation holds a lower proportion in the range of 40~50° in the rail steels 1917 and 1919.

## 1. Introduction

Rail steels are the main component of railway construction, and their consumption is extremely large, thus, the self-production of rail steels has become an inevitable appeal for China’s railway construction. To realize the dream that China’s first railway (Peking-Hankow Railway) construction was completed by China’s self-production of rail steels, the building of Hanyang Iron Works was started in 1890 and put into production in 1894, which was the earliest government-run iron and steel enterprise in the industrial history of modern China and also the only manufacturer of rail steels. The rail steels produced by Hanyang Iron Works in the early period were of poor quality due to the higher content of phosphorus. Therefore, the Hanyang Iron Works carried out large-scale equipment renovations adapted from Bessemer converter steelmaking to Siemens-Martin flat-furnace steelmaking and renewed the rolling equipment from 1905 to 1908. Since then, the control of phosphorus and sulfur elements has been greatly enhanced, and the rolling technology has progressed, thus improving the rail steel quality. Facing the urgent demand for market space in railway construction, Hanyang Iron Works gradually became the main supplier of rail steels to China’s railways after 1908. The good news was that the rail steels of Hanyang Iron Works won the best prize at the Italian World Exhibition in 1911. Moreover, the output of rail steels reached more than 400 tons. However, after a short golden period, Hanyang Iron Works was forced to shut down in 1922 due to a variety of reasons, such as the loss of tariff autonomy, control and suppression by foreign capital and changes in the standard of rail steels. In just over 20 years, the development of the self-production of rail steels manufactured by Hanyang Iron Work represented a particularly difficult situation in the iron and steel industry of modern China [1,2,3,4,5,6,7,8,9,10,11,12].

In this study, a change in the microstructure of rail steels manufactured by Hanyang Iron Works in different years is investigated using a combination of modern characterization methods, including SEM and EBSD, and the effect of chemical composition and microstructural evolution on the rail steels’ performance is discussed, which enables us to review the progress of Hanyang Iron Works and determine the way forward in the development of production technology and overall performance of rail steels.

## 2. Experimental Procedures

The rail steels manufactured by Hanyang Iron Works in 1904, 1911, 1917, 1919 and 1921 were used in this study, hereafter referred to as the rail steels 1904, 1911, 1917, 1919 and 1921. The samples were intercepted to study microstructural evolution according to China Railway Industry Standards TB/T 2344.1-2020 “Steel rails Part 1: 43 kg/m to 75 kg/m steel rails” [13]. The chemical composition of the rail steels 1904, 1911, 1917, 1919 and 1921 was measured using a SPECTRO MAXx spark direct reading spectrometer at room temperature (20 ± 1 °C). The resolutions of the spectrometer are 0.001% for the elements such as carbon, silicon, manganese and copper and 0.0001% for the elements such as phosphorus, sulfur, chromium and nickel. In addition, the average values obtained from three measurements were listed. Moreover, the U71Mn steel (0.65~0.80 %C, 0.15~0.58 % Si, 0.70~1.20 %Mn and other trace elements corresponding to TB/T 2344.1-2020) was selected as a standard sample. In addition, the gas element content was conducted using a LECO CS844 analyzer at room temperature. The microstructures of the rail steels 1904, 1911, 1917, 1919, 1921 were respectively observed by a LEIKA DM2700M optical microscope (OM) and a ZEISS Sigma 300 field emission scanning electron microscope (SEM) equipped with electron backscattered diffraction (EBSD). The samples for OM and SEM observations were prepared by mechanical grinding with sandpaper, mechanical polishing and then etching using a solution of 4% nitric acid alcohol. Besides, the samples for EBSD analyses were mechanically ground and electrolytically polished using a solution of 10% perchloric acid alcohol. The EBSD measurement was carried out using a C-Nano EBSD detector, which is operated at 20 kV with a scanning step size of 0.5 μm. The grain size, grain boundary proportion, grain boundary misorientation, Schmid factor and local misorientation were analyzed from the obtained EBSD results using AZtecCrystal software. The average values such as grain diameter and grain boundary proportion in this study were summarized from three or more maps in different EBSD detection areas.

## 3. Results and Discussion

### 3.1. Chemical Composition

Table 1 shows the chemical composition of the rail steels 1904, 1911, 1917, 1919 and 1921. It was found that the carbon content in the rail steel 1904 was 0.15%, which belongs to a class of low carbon steels. Moreover, the phosphorus content reached up to 0.173%. The Bessemer converter was used for steelmaking in the early years of Hanyang Iron Works, and thus, the phosphorus was not effective to remove, resulting in a greater brittleness and poor quality of rail steels. To resolve these problems, Hanyang Iron Works underwent technology upgrading and equipment replacement from 1905 to 1908 that introduced Siemens-Martin flat-furnace to achieve the improvement in the rail steel quality. After that, the carbon content increased, and the phosphorus content decreased in the rail steels 1911, 1917, 1919 and 1921. Furthermore, it is a significant feature of the rail steels manufactured after the innovation that the copper content rose to approximately 0.50%, which effectively improves the corrosion resistance. On the other hand, the higher chromium content exists in rail steels manufactured after the innovation, resulting in higher wear resistance, corrosion resistance and hardenability.

### 3.2. Microstructures

**Figure 1:**
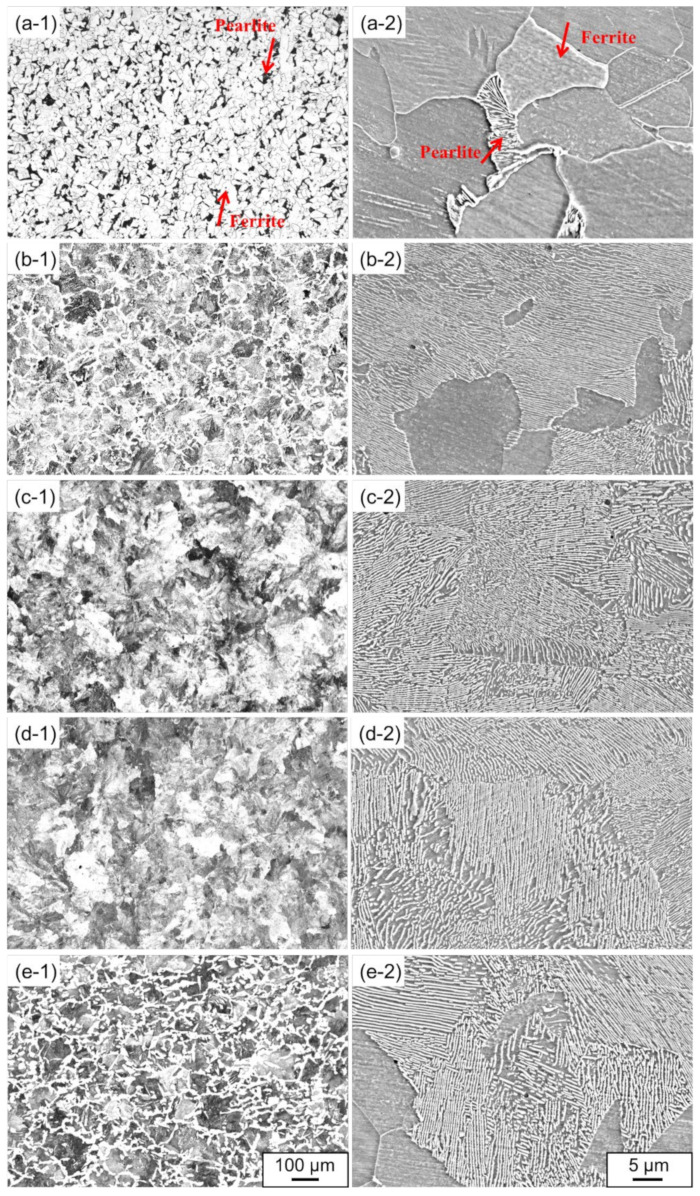
OM and SEM images of microstructures for the rail steels: (**a**) 1904, (**b**) 1911, (**c**) 1917, (**d**) 1919, (**e**) 1921, respectively as shown in (-1) and (-2).

**Figure 2:**
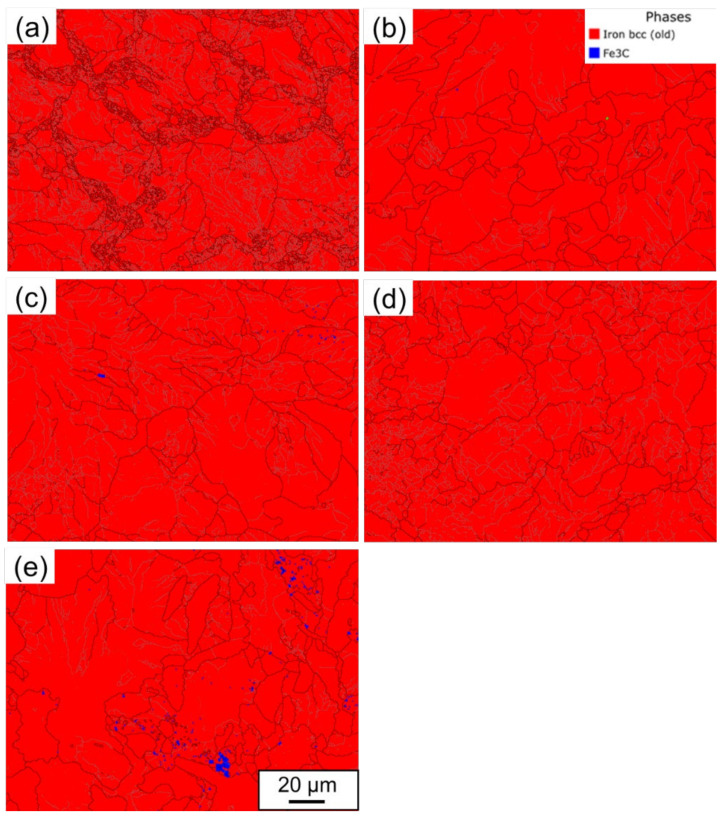
Phase images of microstructures for the rail steels: (**a**) 1904, (**b**) 1911, (**c**) 1917, (**d**) 1919, (**e**) 1921.

Figure 1 shows the OM and SEM images of the microstructures in the rail steels (a) 1904, (b) 1911, (c) 1917, (d) 1919, (e) 1921, respectively as shown in (-1) and (-2). In the OM images, the white and black (or grey) represent the ferrite and pearlite indicated by the red arrows, respectively; however, a portion of pearlite is white owing to the different orientations of pearlite in the observation plane. In Figure 1(a-1),(a-2), the microstructure is ferrite with a small amount of pearlite in the rail steel 1904. Besides, the microstructure is reticular ferrite and pearlite in the rail steels 1911 and 1921 in Figure 1(b-1),(e-1),(b-2),(e-2). Furthermore, the rail steels 1917 and 1919 exhibit full pearlite microstructure, and the pearlite microstructures are accompanied by smaller lamellar spacing, as shown in the high magnification images of Figure 1(c-2),(d-2). The change in the ferrite morphology and pearlite amount is related to the increase in carbon content, which can lead to high hardness and wear resistance for the rail steels. Furthermore, it can be seen in Figure 1(e-2) that the amount of spherical pearlite increases, which is presumably caused by the higher chromium content in the rail steel 1921.

Figure 2 shows phase + grain boundary maps of the microstructures for the rail steels (a) 1904, (b) 1911, (c) 1917, (d) 1919, (e) 1921, which are obtained from EBSD measurements. The bcc and Fe_3_C are respectively shown as red and blue in the phase + grain boundary maps. In Figure 2, it can be seen that all the rail steels 1904, 1911, 1917, 1919 and 1921 hold bcc structures. Furthermore, the amount of Fe_3_C precipitation in the rail steel 1921 was the highest, which can increase the strength of the rail steel 1921. It is presumably related to the highest chromium content and heat treatment. Unfortunately, it is difficult to detect the Fe_3_C phase in the rail steel 1904 under the EBSD experimental magnification in this study, presumably due to the lower amount of pearlite clusters with small size in the microstructure.

**Figure 3:**
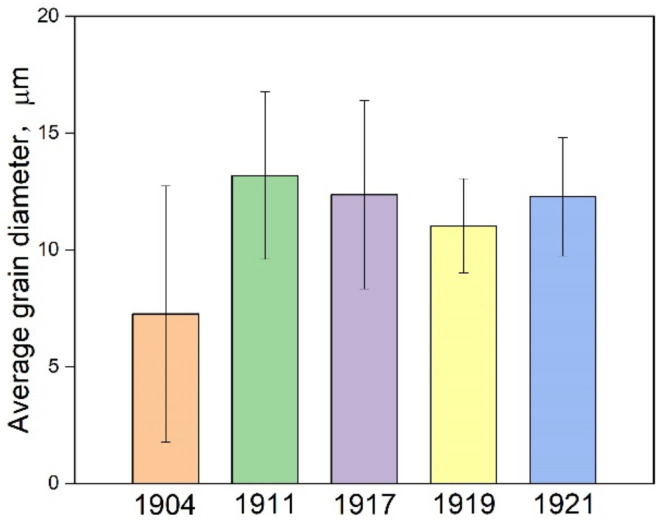
Diagram of average grain diameter for the rail steels.

Figure 3 shows the average grain diameter of the rail steels 1904, 1911, 1917, 1919 and 1921, where the average grain diameter of the rail steel 1904 represents the average values of the ferrite and pearlite cluster microstructures, while it demonstrates the average values of pearlite clusters for other rail steels. In Figure 3, it can be seen that the average grain diameter of the rail steel 1904 is approximately 7.25 μm, which was the smallest of all rail steels manufactured by Hanyang Iron Works, owing to pearlite clusters with small size in the microstructure. The average grain diameter exhibits a decreasing trend compared with the rail steels 1911 and 1921, which have the microstructures of reticular ferrite and pearlite. Moreover, a similar trend exists in the case of the microstructures of full pearlite in the rail steels 1917 and 1919. In other words, after renovation and expansion in 1908, the average grain diameter had a decreasing trend for the rail steels 1911, 1917 and 1919. Nevertheless, the rail steel 1921 exhibited a larger average grain diameter than the rail steel 1919, which may be attributed to them being on the verge of bankruptcy. The grain refinement in the rail steels suggests that Hanyang Iron Works made a great improvement in rolling equipment, technology and experience.

**Figure 4:**
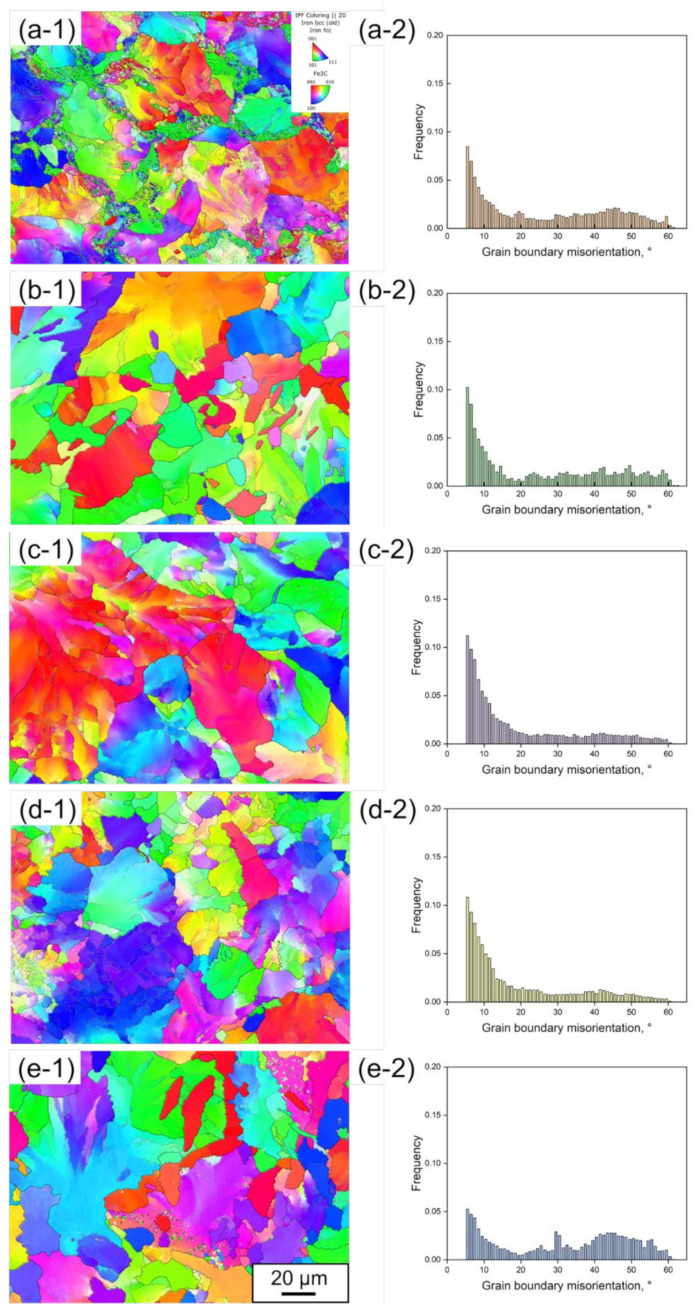
IPF + grain boundary maps and corresponding distribution diagrams of grain boundary misorientation for the rail steels: (**a**) 1904, (**b**) 1911, (**c**) 1917, (**d**) 1919, (**e**) 1921, noted as (-1) and (-2).

Figure 4 shows Inverse Pole Figure (IPF) + grain boundary maps and the corresponding distribution diagrams of grain boundary misorientation for the rail steels (a) 1904, (b) 1911, (c) 1917, (d) 1919, (e) 1921, noted as (-1) and (-2). In Figure 4(c-2),(d-2), the distribution of grain boundary misorientation holds almost the same for the rail steels 1917 and 1919, where high grain boundary misorientation is concentrated in the range of 5~10°. Comparatively speaking, the grain boundary misorientation for the rail steels 1904, 1911 and 1921 is distributed in the range of 5~10° and 40~50°, as shown in Figure 4(a-2),(b-2),(e-2). If the grain boundary misorientation between one grain and the adjacent grains is larger, the stresses are more likely to be concentrated within this grain during plastic deformation, improving the crack nucleation.

**Figure 5:**
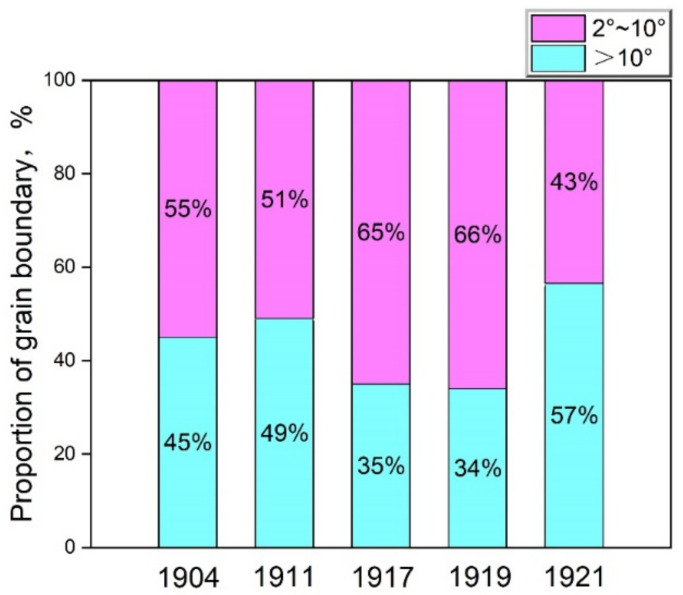
Proportion diagram of grain boundary for the rail steels.

Figure 5 shows the proportion of the grain boundary in the rail steels 1904, 1911, 1917, 1919 and 1921, where blue and pink respectively represent the high angle grain boundaries (HAGBs, > 10°) and low angle grain boundaries (LAGBs, 2~10°) obtained from three or more IPF + grain boundary maps. The proportion of LAGBs for the rail steels 1904, 1911 and 1921 is around 50%, whereas that for the rail steels 1917 and 1919 reaches above 65%. The high content of LAGBs can weaken the conflict effect to the deformation of dislocation slip leading to strengthening. It is also indicated that the rolling aspect advanced constantly after 1908. 

**Figure 6:**
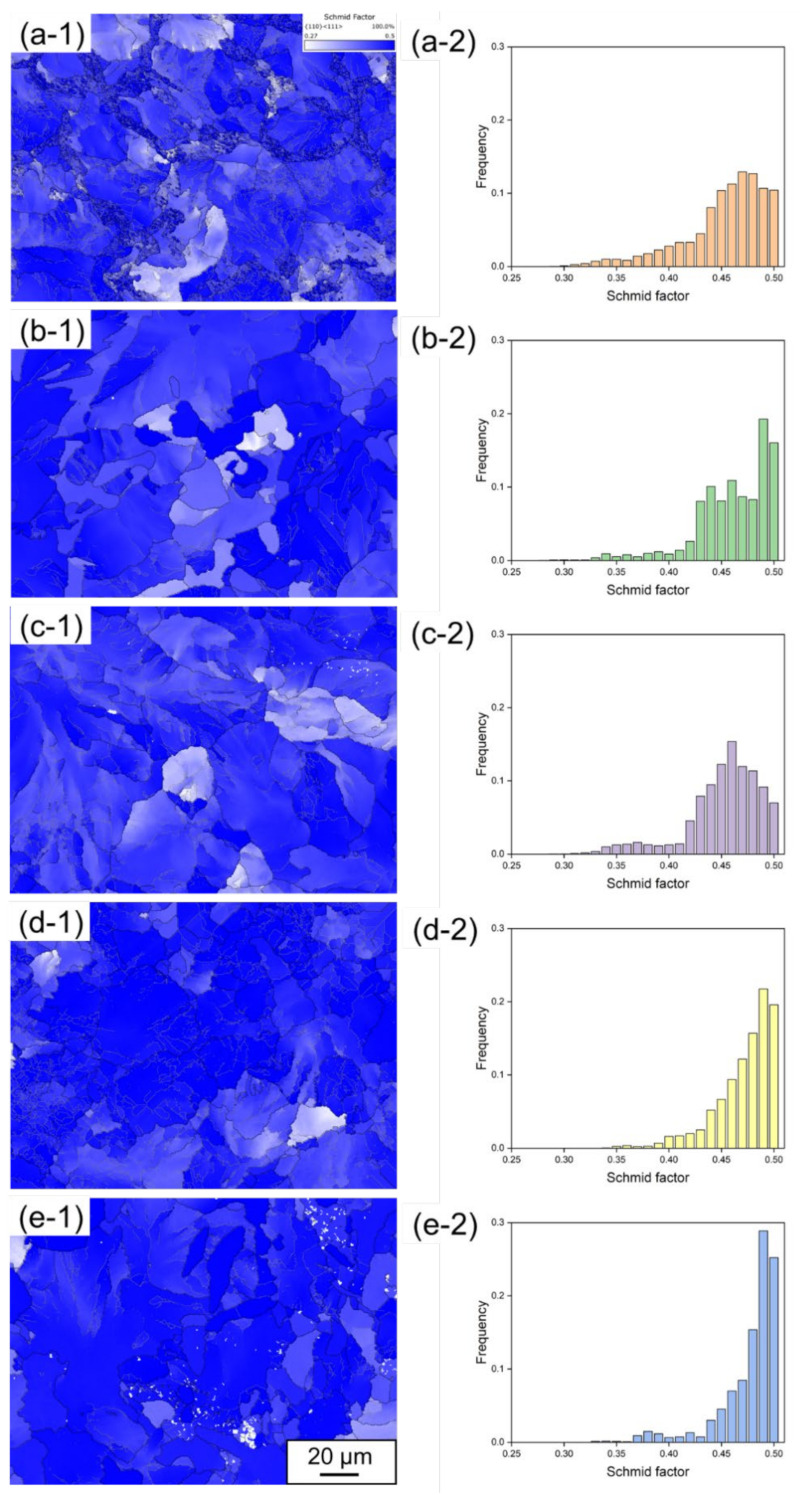
Schmid factor + grain boundary maps and corresponding distribution diagrams of Schmid factor for the rail steels: (**a**) 1904, (**b**) 1911, (**c**) 1917, (**d**) 1919, (**e**) 1921, noted as (-1) and (-2).

Figure 6 shows Schmid factor + grain boundary maps and corresponding distribution diagrams of Schmid factor in slip system {110} <111> for the rail steels (a) 1904, (b) 1911, (c) 1917, (d) 1919 and (e) 1921, noted as (-1) and (-2). The average values of the Schmid factor were 0.45, 0.46, 0.45, 0.47 and 0.47 for the rail steels 1904, 1911, 1917, 1919 and 1921, respectively. Moreover, Schmid factor holds a high frequency in the range of 0.45~0.50. The results suggest that the high Schmid factor values probably tend to initiate the slip system {110} <111>, leading to form {110} <111> texture and thus anisotropy of mechanical properties for the rail steels.

**Figure 7:**
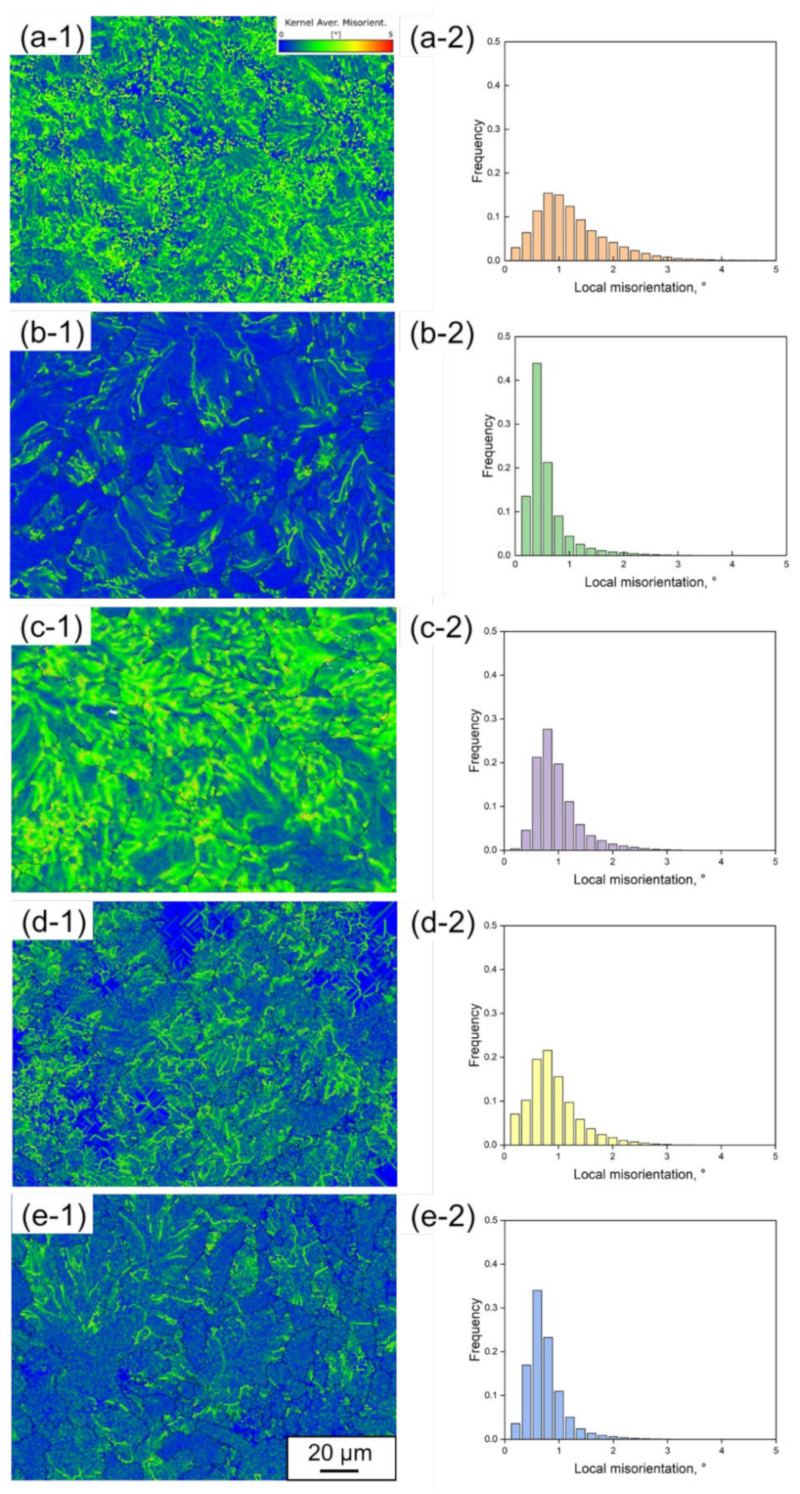
KAM + grain boundary maps and corresponding distribution diagrams of local misorientation for the rail steels: (**a**) 1904, (**b**) 1911, (**c**) 1917, (**d**) 1919, (**e**) 1921, referred to as (-1) and (-2).

Figure 7 is the Kernel Average Misorientation (KAM) + grain boundary maps and the corresponding distribution diagrams of local misorientation for rail steels (a) 1904, (b) 1911, (c) 1917, (d) 1919 and (e) 1921, referred to as (-1) and (-2). The average values of local misorientation are 1.1, 0.5, 0.9, 0.8 and 0.6, respectively, for the rail steels 1904, 1911, 1917, 1919 and 1921, which suggests that the higher average value corresponds to larger plastic deformation. The distribution diagrams show that the local misorientation is mostly lower than 1° for full pearlite in the rail steels 1917 and 1919, corresponding to lower geometrically necessary dislocations density, indicating that the larger strain should be generated during plastic deformation.

As mentioned above, the rail steel 1904 produced by the Bessemer converter exhibits low carbon content and high phosphorus content, having the microstructure of ferrite with a small amount of pearlite, resulting in poor quality and performance. From 1905 to 1908, the steelmaking equipment was replaced by Siemens-Martin flat-furnace, and the open-train mill was renewed. After innovation, the microstructure of pearlite became refined for the rail steels and had a high proportion of low-angle grain boundaries and local misorientation with the angle lower than 1°, and a low proportion of grain boundary misorientation in the range of 40~50°, leading to excellent mechanical properties. The microstructural evolution is presumably contributed to by the rolling technologies, such as rolling temperature, rolling pass, rolling speed, rolling load, etc. Although the Hanyang Iron Works’ equipment and technology fell into disuse in the modern era, the effect of microstructural evolution on the overall performance is the cornerstone and guidance of Chinese rail steels development.

## 4. Conclusions

The change in the microstructures of rail steels manufactured by Hanyang Iron Works can be summarized as follows:

(1). The rail steel 1904 has ferrite with a small amount of pearlite, the rail steels 1911 and 1921 have reticular ferrite and pearlite and the rail steels 1917 and 1919 have full pearlite.
(2). The pearlite grain gradually refines, and the average grain diameter of pearlite was smallest in the rail steel 1919.
(3). The rail steels 1917 and 1919 hold a higher proportion of low-angle grain boundaries and local misorientation with angles lower than 1°, while a lower proportion of grain boundary misorientation was in the range of 40~50°.

## Figures and Tables

**Table 1:** Chemical composition of the rail steels manufactured by Hanyang Iron Works (mass fraction/%).

Year	C	Si	Mn	P	S	Cr	Ni	Cu
1904	0.15	0.02	0.61	0.173	0.049	0.006	0.033	0.16
1911	0.53	0.08	0.65	0.072	0.084	0.035	0.029	0.45
1917	0.68	0.08	0.52	0.027	0.045	0.027	0.021	0.58
1919	0.67	0.08	0.50	0.026	0.034	0.027	0.022	0.58
1921	0.49	0.04	0.46	0.036	0.038	0.049	0.032	0.54

## References

[1] Liu L. (2019). A study on the harm of sodiers and the railway crisis in Beiyang period: Using Hankou-Beijing railway as an example. J. Southwest Jiaotong Univ..

[2] Ding Y. (2004). Zhang Zhi-dong and the railway construction of China. Tangdu J..

[3] Zhu C. (2003). Zhang Zhi-dong and the construction of Luhan railroad. J. Guangxi Norm. Univ..

[4] Feuerwerker A. (1990). China’s Early Industrialization: Sheng Hsuan-Huai (1844–1916) and Mandarin Enterprise.

[5] Fang Y., Qian W. (2005). The Hanyang Iron Works and China’s early railway construction: The characters of China’s early iron and steel industrialization. Chin. J. Hist. Sci. Technol..

[6] Zhang S. (2018). Abandoning shell furnace in Hanyang Iron plant revisted. J. Hubei Polytech. Univ..

[7] Li H. (2010). The Study on the Developmental History of Iron and Steel Industry in Modern China (1840~1927).

[8] Chen D. (2013). The Study on Railway Rail Production in Late Qing Dynasty (1889–1911).

[9] Dai L. (2005). The Hankou-Daye-Pingxiang company’s steel sales and China’s modern steel market (1908–1927). Mod. Chin. Hist. Stud..

[10] Xiao Z. (1979). Overview of the production and use of steel rails in China and developments in foreign rail production. Wuhan Iron Steel Corp. Technol..

[11] Ren Y., Zhang Y., Li Y., Wang W. (2021). Comparative study on rail performance over a century. China Railw..

[12] Fang Y., Dong H. (2020). Modern Chinese Steel Rails: Technical History and Cultural Relic.

[13] National Railway Administration of the People’s Republic of China (2020). Rails—Part 1: 43 kg/m~75 kg/m Rails.

